# Identification of Activated Cdc42-Associated Kinase Inhibitors as Potential Anticancer Agents Using Pharmacoinformatic Approaches

**DOI:** 10.3390/biom13020217

**Published:** 2023-01-22

**Authors:** Vikas Kumar, Raj Kumar, Shraddha Parate, Danishuddin, Gihwan Lee, Moonhyuk Kwon, Seong-Hee Jeong, Hyeon-Su Ro, Keun Woo Lee, Seon-Won Kim

**Affiliations:** 1 Department of Bio & Medical Big Data (BK4 Program), Division of Life Science, Research Institute of Natural Science (RINS), Gyeongsang National University (GNU), 501 Jinju-daero, Jinju 52828, Republic of Korea; 2 ANGEL i-Drug Design (AiDD), Jinju 52828, Republic of Korea; 3 Department of Biotechnology and Bioinformatics, Jaypee University of Information Technology, Waknaghat, Solan 173 234, Himachal Pradesh, India; 4 Division of Applied Life Science, Plant Molecular Biology and Biotechnology Research Center (PMBBRC), Gyeongsang National University (GNU), 501 Jinju-daero, Jinju 52828, Republic of Korea; 5 Department of Chemistry and Molecular Biology, University of Gothenburg, 405 30 Göteborg, Sweden; 6 Division of Applied Life Science (BK21 Four), ABC-RLRC, PMBBRC, Gyeongsang National University, Jinju 52828, Republic of Korea

**Keywords:** ACK1, pharmacophore modeling, docking, molecular dynamics simulations, cancer, inhibitor

## Abstract

Background: Activated Cdc42-associated kinase (ACK1) is essential for numerous cellular functions, such as growth, proliferation, and migration. ACK1 signaling occurs through multiple receptor tyrosine kinases; therefore, its inhibition can provide effective antiproliferative effects against multiple human cancers. A number of ACK1-specific inhibitors were designed and discovered in the previous decade, but none have reached the clinic. Potent and selective ACK1 inhibitors are urgently needed. Methods: In the present investigation, the pharmacophore model (PM) was rationally built utilizing two distinct inhibitors coupled with ACK1 crystal structures. The generated PM was utilized to screen the drug-like database generated from the four chemical databases. The binding mode of pharmacophore-mapped compounds was predicted using a molecular docking (MD) study. The selected hit-protein complexes from MD were studied under all-atom molecular dynamics simulations (MDS) for 500 ns. The obtained trajectories were ranked using binding free energy calculations (ΔG kJ/mol) and Gibb’s free energy landscape. Results: Our results indicate that the three hit compounds displayed higher binding affinity toward ACK1 when compared with the known multi-kinase inhibitor dasatinib. The inter-molecular interactions of Hit1 and Hit3 reveal that compounds form desirable hydrogen bond interactions with gatekeeper T205, hinge region A208, and DFG motif D270. As a result, we anticipate that the proposed scaffolds might help in the design of promising selective ACK1 inhibitors.

## 1. Introduction

**Figure 1:**
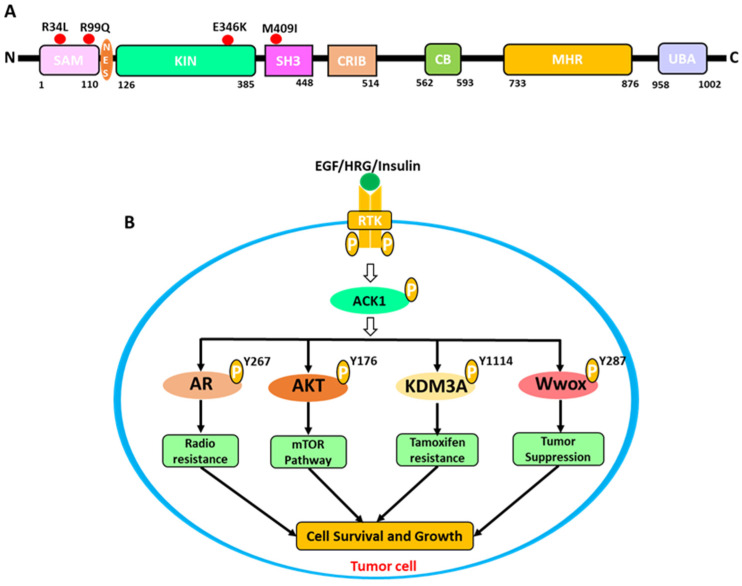
(**A**) Schematic representation of the ACK1 protein domains. The mutations were highlighted with a red circle; among them, the E346K mutation was significantly increased in ovarian cancer. (**B**) The signaling network of ACK1. The activated receptor tyrosine kinase pathway induces cell survival and proliferation by activating ACK1 and its downstream signaling patterners.

The tyrosine kinases are the key regulators of cytoplasmic signaling cascades. The dysregulation of their activity by overexpression or point mutation may result in hyperactivation [1]. Continuous activation of tyrosine kinases can lead to increased cell growth, cell migration, suppression of apoptosis, angiogenesis, invasion, etc. [2,3]. As a result, tyrosine kinases have been classified as distinct drug targets for anti-cancer therapies. Tyrosine kinases are mainly of two types: receptor and non-receptor tyrosine kinases [4]. ACK1 is a member of the non-receptor tyrosine kinases (NRTK) family [5]. The TNK2 gene on chromosome 3q29 codes for the ACK1 protein, which is linked to various human cancer [6,7,8]. ACK1 is a multidomain protein of approximately 140 kDa in weight and 1038 amino acids long (Figure 1A) [9]. It is usually expressed in mammals, with the greatest levels seen in the spleen, thymus, and brain [8]. The role of ACK1 has been well established in cell survival, proliferation, migration, and brain development [5,9]. Moreover, several pre-clinical studies have reported that ACK1 overexpression is responsible for various cancers, such as prostate, breast, pancreatic, ovarian, lung, gastric, hepatocellular, and renal carcinoma [10,11,12]. ACK1 acts as an oncogenic kinase by phosphorylating and activating critical survival-promoting kinase receptors on various tyrosine kinase residues (Figure 1B).

In cancer cells, ACK1 stimulates AKT by phosphorylating it at Y176, which promotes AKT downstream signaling and contributes to cell survival and proliferation [13]. Activated ACK1 also contributes to stopping the function of the important tumor suppression protein Wwox by phosphorylating it at Y287 [1]. The androgen receptor phosphorylation by ACK1 at Y267 and Y363 indorses PI3K independent activation and the development of prostate cancer [14]. Since the ACK1 role is linked to numerous malignancies, inhibiting this protein is a prospective therapy option for a variety of tumors. To date, various research and industrial groups have identified six different types of selective inhibitors against ACK1, which have shown anticancer effects [15]. The multi-kinase FDA-approved inhibitors have also been tested against ACK1. The most active multi-kinase inhibitor reported against ACK1 in an in vitro study was dasatinib [16]. KRCA-0008, another multi-kinase inhibitor with significant inhibitory efficacy against ACK1 and ALK proteins, is currently being investigated [17]. AIM-100 is the first ACK1 inhibitor that has been studied the most. It was discovered through a high-throughput screening assay [18]. The inhibitors identified against ACK1 to date have not progressed to clinics due to off-target effects and poor pharmacokinetic properties [9,15]. Therefore, there is an urgent need for novel therapeutic options against ACK1-related malignancies. Computer-aided drug design is the first step in rational drug design and is nowadays used commonly for the identification of potential scaffolds against many therapeutic proteins [19,20]. Correspondingly, in our study, we applied a series of computational approaches for the identification of novel drug-like ACK1-selective scaffolds. First of all, the available inhibitor-bound structures were collected from PDB and were subsequently analyzed for the protein–ligand interaction. Based on key molecular interactions with the ACK1 protein active site residues, two structures bound with different inhibitors were further selected for structure-based pharmacophore modeling (PM). All generated pharmacophores were merged, and a dynamic pharmacophore was generated. The chosen PM was validated and used to screen the drug-like database. The MD study was used to predict the binding potential of the chosen compounds. Further, the validation of docking results was done using MDS studies. Finally, three hit candidates with unique scaffolds were identified as potentially effective ACK1 inhibitors based on binding free energy and critical molecular interactions. Our study provides an integrated computational methodology based rational approach for the identification of hit candidates. However, further experimental validation of the identified hit candidates is warranted.

## 2. Materials and Methods

### 2.1. Structure-Based Pharmacophore Generation

Six inhibitor-bound crystal structures of human ACK1 have been discovered so far, according to a protein data bank search (PDB, www.rcsb.org/pdb (accessed on 10 November 2021). All structures were retrieved from the protein data bank and processed in Discovery Studio. We chose two crystal structures, 1U4D and 3EQR, based on their level of resolution and inhibitor interactions with active site residues, to develop structure-based ACK1 PMs [21,22]. The *Receptor–Ligand Pharmacophore Generation* protocol of DS was utilized to generate hypotheses with default parameters. Subsequently, the generated models from these structures were merged independently, and single hypotheses were retained from each structure. Finally, these two hypotheses were superimposed and placed in the active site of ACK1. Rationally, chemical features mapping with the most important residues was selected for the generation of the final hypothesis [23,24].

### 2.2. Validation of Pharmacophore

The efficacy of the pharmacophore to differentiate among active and inactive ACK1 inhibitors was analyzed using the Guner–Henery method [25]. In this method, a decoy test set was generated with both active and inactive compounds known experimentally against ACK1 [26,27]. The decoy data set was then screened on our PM using DS studio. The obtained screening data were used to calculate various important parameters by solving the Guner–Henery equation [25].

(1) $$GH=\left(\frac{Ha\;\left(3A+Ht\right)}{4HtA}\right)\times \left(1-\frac{Ht-Ha}{D-A}\right)$$

(2) $$EF=\frac{Ha/Ht}{A/D}\;$$

A GH score value approaching 1 indicates an ideal pharmacophore hypothesis for chemical database screening [26,27].

### 2.3. Virtual Screening

Chemical databases, such as ZINC, NCI, ASINEX, and Princeton, were considered for pharmacophore-based screening. Selected databases include thousands of compounds, some of which may have poor drug-like and pharmacokinetic characteristics, making screening all compounds in these databases worthless. Therefore, these databases were filtered by various filters, such as ROF and ADMET Descriptors in DS respectively [28,29]. Subsequently, the selected PM was provided as input, and the drug-like database screening was performed using DS with Best/Flexible option [30].

### 2.4. Molecular Docking

The Genetic Optimization of Ligand Docking (GOLD v5.2.2) package was used for the docking study [31]. The GOLD program offers full ligand flexibility and partial protein flexibility, resulting in more consistent docking simulations. The 3D structure of ACK1 (PDB ID: 1U4D) was selected for MD studies [21]. The structure was downloaded from the PDB databank (https://www.rcsb.org/ (accessed on 15 January 2022) and prepared for MD in DS. The structure was obtained in dimer form; therefore, Chain A and other unwanted molecules were deleted. Subsequently, the structure was cleaned using the *Clean Protein* module of DS, and missing residues were added. The docking site was defined around the PDB 1U4D-bound inhibitor. The cocrystal ligand and multi-kinase inhibitor dasatinib were used as reference compounds for docking analysis. The Goldscore and Chemscore were used to rank the compounds [32,33]. For each compound, 10 poses were produced. The best conformer was selected on the basis of consensus scoring and a better docking score than the two REF compounds. The best pose was further visualized in DS for crucial polar and non-polar interactions with the active site residues of ACK1 [34].

### 2.5. Molecular Dynamics Simultaions

The MDS for all the selected ACK1-inhibitor complexes were performed using the *Groningen Machine* for *Chemical Simulation* (GROMACS *v*2022.2) [35,36]. The topology file for protein was generated with the CHARMm27 forcefield in GROMACS, and ligand files were generated from SwisParam [37,38]. The MDS were run in a dodecahedron box with a TIP3P water model. The neutralization of the simulation system of each protein–ligand complex was performed with appropriate Na^+^/Cl^−^ ions. The energy minimization of the system was performed with 10 kJ/mol to avoid steric collision and inappropriate contact. In the equilibration step, temperature and pressure equilibration were performed under NVT and NPT ensembles for 1000 ps at 300K. During the equilibration process, the protein backbone was restricted, whereas the solvent molecules and ions were permitted to circulate. The MDS of each system was performed under periodic boundary conditions for 500 ns. section may be divided into subheadings. It should provide a concise and precise description of the experimental results and their interpretation, as well as the experimental conclusions that can be drawn.

### 2.6. Binding Free Energy Analysis

The binding affinity calculations can provide mechanistic insights into protein–ligand interaction and therefore can play a crucial role in the selection of high affinity candidates against the target protein [39,40]. In the present study, ΔG was calculated for simulated ACK1-inhibitor complexes using the molecular mechanics Poisson-Boltzmann surface area (MM-PBSA) approach [41]. From the 500 ns simulation trajectory, a total of 40 snapshots were extracted for free energy calculation. The analyses were performed using the GROMACS plugin tool g_mmpbsa [42]. The different components involved in protein–ligand interactions, such as electrostatic, polar, and non-polar solvation, were calculated. The binding free energy of the ACK1-ligand complex in a solvent was calculated using the following equations:

(3) ΔGbinding=Gcomplex−(Gprotein+Gligand)             

where G_complex_ is the total protein–ligand complex energy, G_protein_ and G_ligand_ denoting individual energy components. The per residue contribution of each residue was also calculated using the MmPbSaDecomp py python script. A complete description of the method can be found elsewhere [42].

### 2.7. Principal Component and Free Energy Landscape Analyses

MDS trajectories were utilized for the analyses of pattern recognition in protein movements using the GROMACS tool “gmx_covar” [35]. In principal component analysis (PCA), eigenvectors and eigenvalues were first computed using the covariance matrix [43]. The larger the eigenvalue of the corresponding eigenvector, the higher the motion for this eigenvector coordinate. The 2D plotting of two different eigenvectors is produced using the “gmx_anaeig” tool. Moreover, the free energy landscape was also studied using the Gibbs free energy landscape of the top two eigenvectors using “gmx_sham” [35].

## 3. Results

**Figure 2:**
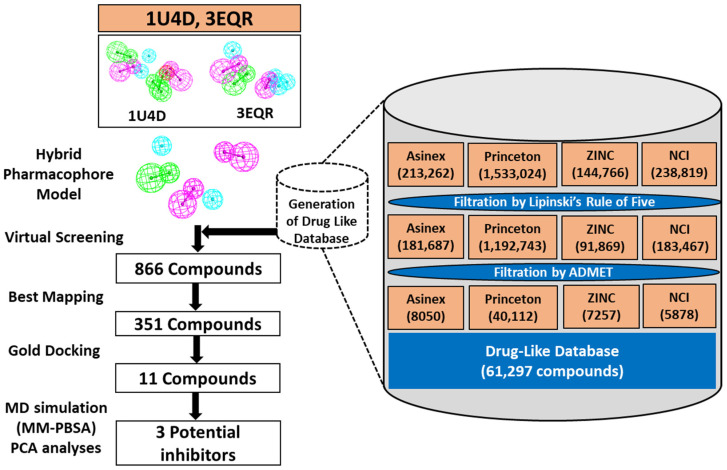
The workflow used in this study for the identification of potential ACK1 inhibitors. Generation of pharmacophores from two different structures. The drug-like database generation step is shown on the right side.

A generalized overview of the study is provided in Figure 2, and the results are explained in detail in the sections below.

### 3.1. Active Site Analysis of ACK1

The structural details reveal that the active site of the ACK1 is surrounded by the gatekeeper (T205), hinge region (206–210), glycine-rich loop (133–138), αC helix (251–257), and DFG (Asp-Phe-Gly) motif (270–272) [21,44]. A detailed observation of the residues targeted by the 3D structure-bound inhibitors via hydrogen bond is summarized in Table 1. The analysis revealed that hinge region residue A208 was targeted by each inhibitor bound to ACK1 via a hydrogen bond. The gatekeeper residue T205, which connects the N and C terminals of the kinase, was observed to form hydrogen bond interactions in two structures: 3EQR and 3EQP bound inhibitor [22]. The role of T205 was reported to enhance ACK1 inhibition in in vitro cellular assays. Therefore, targeting T205 via hydrogen bonds is a desirable characteristic of future ACK1 inhibitors. The conserved DFG motif residue D270, part of the activation loop, was observed to form a hydrogen bond with 1U4D and 5ZXB bound inhibitors [21,45]. This analysis motivated us to use the knowledge of two different protein–inhibitor complexes that target three key residues via hydrogen bonds, A208, T205, and D270, to build a hypothesis that can map potential inhibitors that should target the aforementioned residues [21,22,45]. Therefore, 1U4D and 3EQR were selected rationally for PM generation.

### 3.2. Structure-Based Pharmacophore Modeling

**Figure 3:**
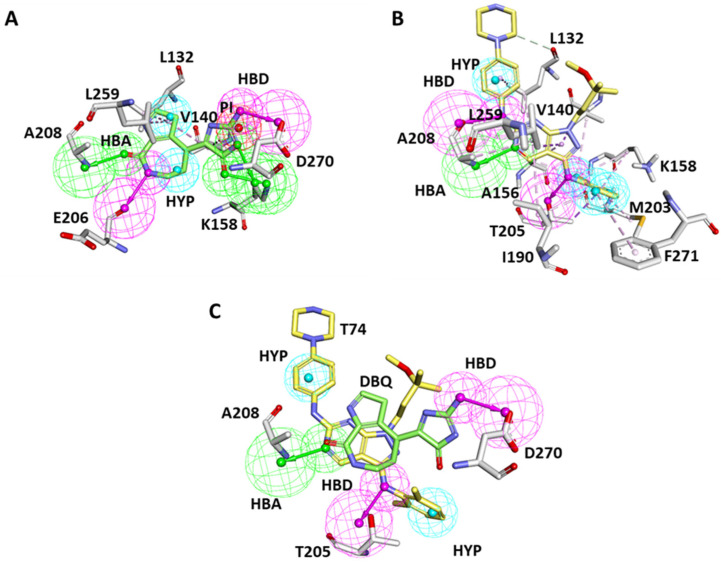
Pharmacophore mapping with active site residues of ACK1 (**A**) PDB id: 1U4D and (**B**) 3EQR overlaid on Pharm A and Pharma B, respectively. The co-crystalized ligands DBQ and T74 are shown with green and yellow sticks. The active site residues are shown with gray sticks. (**C**) Final hybrid pharmacophore model. The key residues A208, T205, and D270 mapped with hydrogen bond acceptor and donor features are shown.

The debromohymenialdisine (DBQ) and T74 ligand-bound crystal structures of ACK1; 1U4D and 3EQR were obtained from the PDB database. The *Receptor–ligand Pharmacophore Generation* module of Discovery Studio (DS) *v*19 was utilized for pharmacophore generation [48]. A total of twenty pharmacophores were generated, ten from each structure (Tables S1 and S2). The generated PMs were subsequently merged using the *Edit and Cluster* tool available in DS, and a single common feature hypothesis was selected from each PDB (Figure S1A,B). The 1U4D pharmacophore (Pharm A) has eight different features: three hydrogen bond donors (HBD), two hydrogen bond acceptors (HBA), two hydrophobic (HYP), and one ionizable position (PI). The merged hypothesis detailed analysis indicates that the imidazole ring of DBQ accommodated two HBA features mapped with K158 and another HBA feature mapped with DFG motif residue D270. The azepine ring of the DBQ accommodates one HBA and one HBD feature, which complements E206 and A208 (Figure 3A). The hydrophobic feature of the pyrrole ring may interact with L132, V140, and L259. The remaining features, such as HYP and PI features, did not show potential mapping with active site residue and therefore could be removed from the pharmacophore (Figure 3A). The merged hypothesis obtained from the PDB 3EQR (Pharm B) displayed seven features: four HYP, two HBD, and one HBA (Figure 3B). The phenyl ring of T74 was mapped with three hydrophobic features. The nitrogen atom that connects the phenyl and pyrimidine rings displayed the HBD feature, which mapped with gatekeeper residue T205. The pyrimidine ring and its adjacent nitrogen atom connect it with the phenyl ring map with A208 via HBA and HBD features, respectively. The adjacent phenyl ring also accommodates the hydrophobic feature (Figure 3B). Further, the hypotheses were superimposed, and a hybrid PM was generated. Rationally common and unique features were retained only. In the final hypothesis common, HBA features complementary to A208 were retained. However, two unique features complementary to T205 and D270 were retained from the 3EQR and IU4D structures. Two hydrophobic features that may map with residues L132, K158, and M203 were also retained from the 3EQR hypothesis (Figure 3C). Robust PM was designed with the aim of targeting these essential residues. The inhibitors that map with purposed PM may target ACK1 with high specificity.

### 3.3. Validation of Pharmacophore

**Figure 4:**
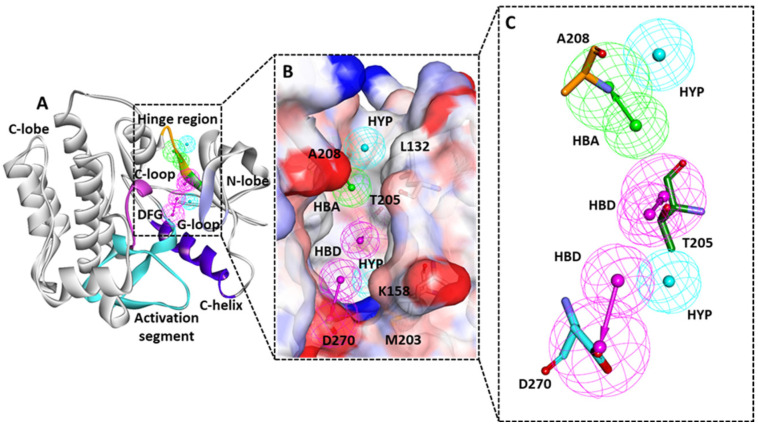
The ACK1 3D structure PDB:1U4D is shown here with ribbon representations. (**A**) The important regions of the ATP-binding site were shown with orange (hinge; 206–210), green (gatekeeper; T205), blue (αC-helix; 168–183), purple (G-rich loop; 133–138), pink (catalytic loop; 251–257), and cyan color (activation segment; 270–300). (**B**) The overlay of pharmacophore inside the active site of protein is shown in A and in the surface representation. (**C**) The hydrogen bond-mapped features are shown in the enlarged view.

The initial validation of the PM was done on the basis of a chemical feature mapping analysis with protein active site residues. The overlay of the final model into the active site of ACK1 confirms the mapping with three key residues, T205, A208, and D270, through the hydrogen bond donor or acceptor feature. The two hydrophobic features retained in the final PM may map with residues L132, K158, and M203 (Figure 4A–C).

Furthermore, a well-known decoy set method approach using the Guner–Henery (GH) method was used for the validation of the PM [25]. For this, a database of experimentally tested 20 active (IC_50_ < 100 nM) and 120 inactive (IC_50_ > 1000 nM) compounds against ACK1 was generated [46,47,49,50,51]. PM was used to perform virtual screening of the drug-like database using the *Ligand Pharmacophore Mapping* protocol of DS. According to our observations, the hypothesis gives GH and EF scores of 0.76 and 5.40, respectively. (Table 2). Additional parameters calculated in this method, such as yields and ratio of actives, false negatives and positives, are shown in Table 2. It can be learned from the validation results that the model displayed great potential in differentiating between active and inactive compounds and therefore can be used for ACK1 inhibitor identification.

### 3.4. Virtual Screening

Four different chemical libraries, Asinex, Princeton, ZINC, and NCI, were considered for pharmacophore-based virtual screening. A total of 61,297 drug-like compounds were obtained after the successful application of drug-like filters from the selected chemical databases (Table 3 and Figure 2).

Subsequently, compounds were imported to DS, and the *Ligand Pharmacophore mapping module* of DS was utilized for virtual screening using PM. Accordingly, 866 compounds were obtained, which were further analyzed in DS for best mapping with PM. Finally, 351 compounds were selected for the MD study with the ACK1 protein. It is noteworthy to mention the significance of PM in the elimination of false binders, reducing 61,297 compounds to 351 (Figure 2).

### 3.5. Molecular Docking

The ACK1 kinase domain 3D structure (PDB id: 1U4D) was taken as a receptor for the docking study [21]. Subsequently, the structure was prepared using DS by deleting the heteroatoms, and chain B was used [52]. The docking site was defined using the *Define and Edit Binding Site* module around the bound inhibitor. The radius of the docking sphere was set at 6 Å and the XYZ coordinates were set as 56.30, 17.14, and 41.12. The MD study was conducted using the GOLD program [31]. During the docking experiment, two known ACK1 inhibitors (cocrystal ligand and dasatinib) were also docked with 351 potential inhibitors. Default docking scores GoldScore and ChemScore were used for the selection of the final hit compounds. Our results demonstrated that the REF drug dasatinib displayed Goldscore 63.78 and Chemscore −25.09 that is greater than the co-crystal ligand Goldscore 45.48 and Chemscore −22.33. Therefore, a docking score greater than dasatinib was used as the first criterion for the selection of potential binders, and a total of 25 compounds were selected. We further analyzed each compound’s molecular interactions with ACK1. As suggested by previous studies, inhibitor interactions with hinge region residue A208, gatekeeper T205, and DFG motif D270 are most important for ACK1 inhibition. The inhibitors that form hydrogen bond interactions with at least two of the mentioned residues were selected. Finally, eleven compounds were obtained. The MD scores and corresponding molecular structures are shown in Table S3 and Figure S2A,B.

### 3.6. Molecular Dynamics Simulations

Initially, a 50 ns molecular dynamics simulation run was conducted for complexes selected from the molecular docking study. The stability of the simulation trajectories was studied using root mean square deviation, fluctuations, and hydrogen bond potential. Further, the ligands were ranked according to binding affinity using the MM-PBSA approach (Table S4). The selected simulation systems were prepared using the GROMACS program and simulated until 500 ns (Table S5). The final compounds were selected on the basis of stability, binding affinity, and key molecular interactions (Table S6).

#### 3.6.1. Stability of the Simulation Complexes

**Figure 5:**
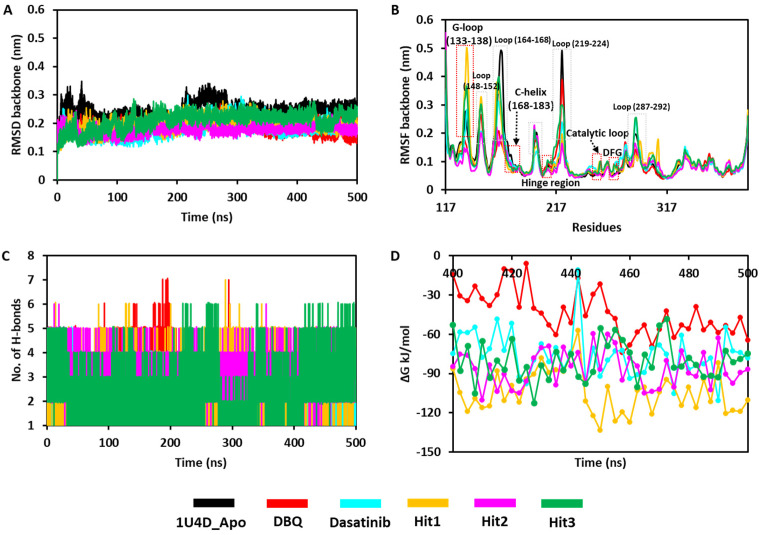
Molecular dynamic simulation analyses. (**A**,**B**) Backbone RMSD and RMSF. (**C**) Number of H-bonds and (**D**) binding free energy plots generated from MM-PBSA calculations.

Figure 5A graphically shows the assessment of the protein backbone atom root mean square deviation (RMSD) analyses. Throughout the simulations, the RMSD values of the Hit compounds and REF inhibitors were observed within a reasonable fluctuation of <0.3 nm (Table S6). The average RMSD value of the PDB structure 1UD4-apo form was 0.24 nm. Interestingly, all the hit compounds, dasatinib and 1U4D-DBQ-bound ACK1 complexes, displayed lower average RMSD values in comparison to the 1U4D-apo form. Hit2 displayed the lowest RMSD values of 0.17 nm, followed by dasatinib and DBQ at 0.18 nm. Hit1 displayed an average RMSD of 0.19 nm, whereas Hit3 displayed an average RMSD of 0.20 nm. The RMSD of the studied ligands is also plotted in Figure S3. The plot indicates that ligands named Hit2 displayed the most stable RMSD throughout the simulation run, followed by Hit3 and Hit1. All the ligands displayed significant fluctuations except Hit2 and Hit3, but interestingly, the average value was observed <0.3 nm (Table S6). The root mean square fluctuation (RMSF) measured protein flexibility and was widely used to analyze the protein’s residual flexibility over the simulation period. The RMSF of the 251 residues of the ACK1 backbone atoms was measured for 500 ns. As shown in Figure 5B, major fluctuations were observed in the loop regions ranging between residue numbers 136 to 167. Interestingly, fewer residual fluctuations were observed in the inhibitor-bound complexes when compared to the apo 1U4D structure. The superimposition of all the simulated complexes revealed that synonymous behavior of protein backbone residues was observed in each system (Figure 5B). Moreover, the catalytically important regions were stable, except for the G-loop region (133–138). Overall, RMSF average values were observed <0.3 nm in all the simulated complexes, indicating the stability of the systems (Table S6). Hydrogen bonds play a significant role as a stabilizing force for a protein–ligand complex. Therefore, the potential of Hit compounds to form hydrogen bonds was analyzed (Figure 5C). Interestingly, we observed that Hit1, Hit2, and Hit3 bound complexes displayed a slightly better potential to form a hydrogen bond with protein residues when compared with dasatinib and DBQ-bound complexes (Table S6). The strong hydrogen bond-forming potential of the Hit compounds indicates that they can bind with proteins effectively and tightly.

#### 3.6.2. Binding Free Energy

The “g_mmpbsa” GROMACS plugin tool was used to predict the binding free energy (ΔG) of the simulated compounds [42]. According to the MM-PBSA calculations, van der Waals interactions were the major contributing force to complex stability. Hit2 displayed the highest van der Waals interaction energy, followed by Hit1, Hit3, dasatinib, and DBQ (Table S7). The electrostatic contribution was highest in Hit3, followed by Hit2, Hit1, DBQ, and Dasatinib. Polar solvation energy contributed positively to the protein–ligand interaction. SASA energy contributes negatively to the interactions. The average ΔG of Hit1 was −104.18 kJ/mol and was found significantly better than other compounds as well as REF inhibitors dasatinib and DBQ, which displayed average ΔG of −75.42 kJ/mol and −43.62 kJ/mol respectively. Hit2 and Hit3 displayed average binding affinity of −86.31 and −80.23 kJ/mol. The detailed contributions of the energies are shown in Table S6 and Figure 5D.

#### 3.6.3. Binding Mode Analysis

**Figure 6:**
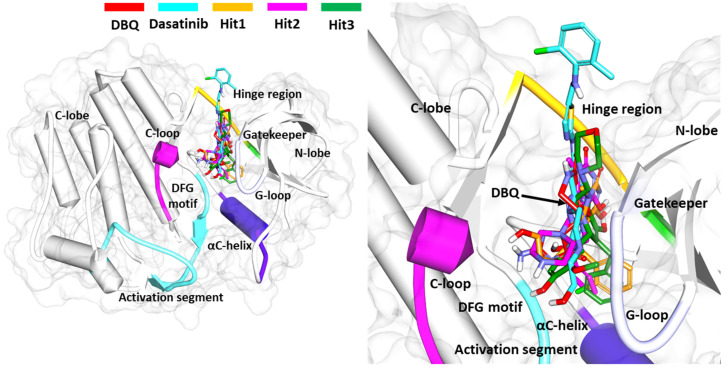
Binding mode of selected Hit compounds. Superimposition of simulated ACK1-inhibitor complexes. The protein is shown in schematic representation with a light gray color surface. The important regions forming the active site were highlighted. The inhibitors are shown in a stick representation with different color codes.

The selected ligands binding with the ACK1 structure were analyzed using the representative structure from the last 5 ns simulation trajectories. The superimposition of all protein–ligand complexes indicates that Hit compounds follow the binding mode similar to crystalized ligand DBQ and the REF inhibitor dasatinib (Figure 6). The active site of the ACK1 protein is located between the smaller N and larger C terminal domains, and it is surrounded by the hinge region, glycine-rich loop, activation loop, and catalytic loop. It is important to note that ACK1 inhibitor-bound crystal structures identified to date target the hinge region residue A208 via hydrogen bond interactions. In vitro studies conducted against ACK1 reveal that gatekeeper residue T205 may play a crucial role in inhibitor activity against ACK1 [22]. The structural details reveal that ACK1 is present in an activated state without phosphorylation; therefore, DFG motif residues, such as D270 and F271, may be important for inhibitor binding. We, therefore, selected only those hit compounds that target A208, T205, and D270 via hydrogen, preferably, or hydrophobic interactions. Our analysis confirms that each selected inhibitor occupies an ATP-binding site similar to the REF inhibitors used in the study.

**Figure 7:**
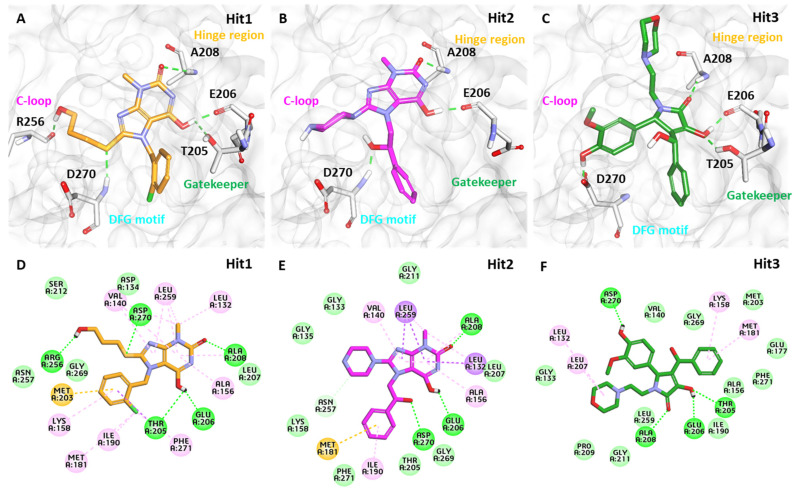
The 3D representation of the molecular interaction of (**A**) Hit1, (**B**) Hit2, and (**C**) Hit3. The hydrogen bonds are shown with green dashed lines. A lower panel (**D**–**F**) displaying detailed molecular interactions in 2D of Hit1, Hit2, and Hit3, respectively.

The average structure of ACK1-Hit1 displayed five hydrogen bond interactions. The purine ring of Hit1 interacts with gatekeeper residue T205, hinge region E206, and A208 via hydrogen bond interactions. The hydroxypropyl sulfanyl chain interacts with catalytic loop residue R256 and DFG motif residue D270 via a hydrogen bond (Figure 7A). The purine and chlorophenyl rings of Hit1 displayed significant pi-alkyl interactions with residues L132, V140, A156, K158, M181, I190, 203, L259, and F271 (Figure 7D). Further, the interactions were also supported via various van der Waals interactions with D134, L207, S212, N257, and G269 (Table 4). The purine ring of Hit2 displayed two hydrogen bonds with residue hinge region residues E206, and A208. Additional hydrogen bonds, as observed with DFG motif residues D270. Unlike Hit1, Hit2 does not display a conventional hydrogen bond with gatekeeper residue T205, but a hydrophobic interaction was observed with the phenyl ring of the hit compound (Figure 7B). The residues G133, G135, K158, T205, L207, G211, N257, G269, and F271 were observed to contribute to van der walls interactions (Figure 7E). The pi-alkyl interactions were observed with residues similar to Hit1, with a slightly lower number (Table 4).

The pyrrole ring of the Hit3 displayed hydrogen bonds with gatekeeper T205, hinge region residue E206, A208, and the methoxyphenyl ring interacts with DFG motif residue D270 (Figure 7C). The hydrophobic interactions in the case of Hit3 were mainly formed by van der Waals interactions with residues G133, V140, A156, I190, E177, M203, P209, G211, L259, G269, and F271 and pi-alkyl interactions with L132, K158, L207, and M181 (Figure 7F). The co-crystalized ligand bound with PDB id 1U4D, named DBQ, was also simulated until 500 ns, and the average structure of the complex was taken from the last 5 ns MDS trajectories. The detailed binding mode of the complex reveals that the inhibitor displayed hydrogen bond interactions with hinge region residue A208 and DFG motif residues D270 (Figure S4A,B). A carbon hydrogen bond was also observed with hinge region residue E206. The crystalized inhibitor failed to show hydrogen bond interaction with residues, such as D134 and K158. Moreover, similar to the crystalized structure, the ACK1-DBQ complex did not display conventional hydrogen bond interaction with gatekeeper T205. The complex displayed seral van der Walls and pi-alkyl interactions with the surrounding active site residues (Table 4). The multi-kinase inhibitor, which showed nanomolar efficacy against ACK1, was also studied under similar conditions, and the average structure of MDS displayed two hydrogen bonds with gatekeeper residue hinge region A208, and DFG motif residue D270 (Figure S5C,D). Further, the REF inhibitor dasatinib forms eight van der Waals interactions and five π-π/ π-alkyl interaction (Table 4). It can be inferred from our analysis that all three-hit compounds form strong hydrogen bond interactions with the key residues of the ACK1 active site (Table 4 and Table 5). Therefore, hit compounds can be further selected for future studies to combat ACK1-related malignancies.

**Figure 8:**
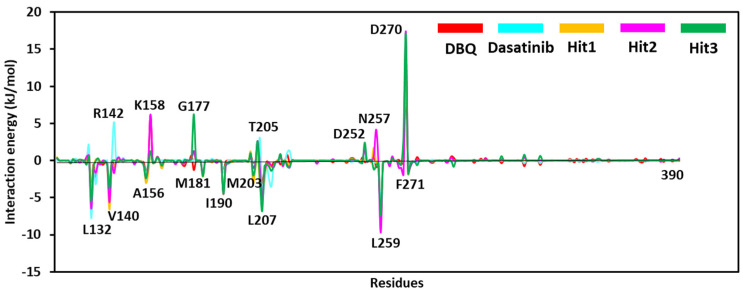
The energy decomposition plot of each residue in the corresponding simulated system was obtained from the MM-PBSA calculation.

The per-residues contribution obtained from free energy calculation can provide more details about protein inhibitor interactions. It can be noticed from Figure 8 that REF inhibitor dasatinib, DBQ, and selected hits target similar residues with different energetics. In particular, L132, V140, A156, M181, I190, M203, L207, L259, and F271 significantly contribute to binding via various hydrophobic interactions. The residues shown on the upper side of the graph, such as R142, K158, G177, T205, N257, and D270, may contribute to polar interactions.

#### 3.6.4. Hydrogen Bond Analysis

**Figure 9:**
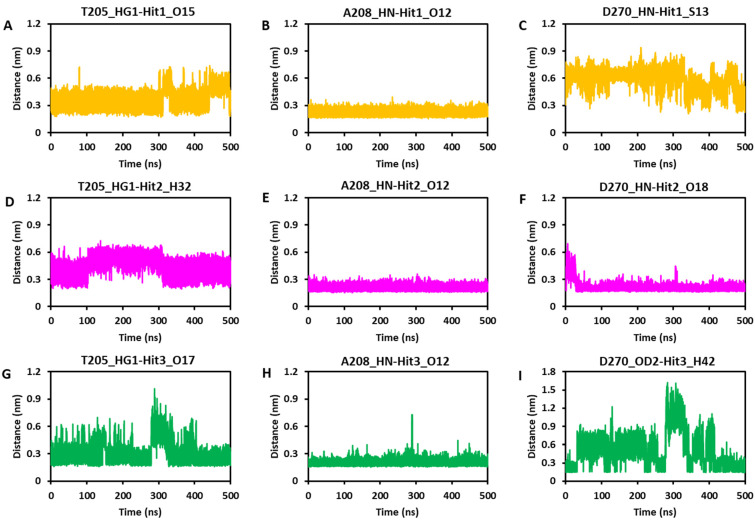
Analyses of Hydrogen bond dynamics. The distance was measured between the ACK1 key residues T205, A208, and D270 with Hit1 (**A**–**C**), Hit2 (**D**–**F**), and Hit3 (**G**–**I**).

Hydrogen bonds (H-bonds) observed from a single snapshot may not always provide significant information; consequently, a complete dynamics analysis of H-bonds can be valuable for studying the stability of hydrogen bonds. As previously stated, the hydrogen bond with important residues T205, A208, and D270 is critical for ACK1 protein inhibition. We plotted the distances between the atoms of the aforementioned protein residues and ligands using the GROMACS program’s ‘gmx hydrogen-bond distance’ function. The average structure reveals that all three hits target A208 and D270 through hydrogen bonding. Hydrogen bonds with T205 were identified exclusively in Hit1 and Hit3 but not in Hit2 and dasatinib (Figure 7 and Table 4). An illustration of the dynamics of the H-bonds for the chosen amino acid residues is provided in Figure 9A–I. It is clear from the graphs that Hit1 displayed hydrogen bond with three key residues, but a hydrogen bond distance with D270 was observed >0.35 nm until 300 ns but after that, the average distance lowered <0.35 nm. Hit2 does not exhibit hydrogen bonding with T205, although the graph indicates that it may form a hydrophobic interaction. Throughout the 500 ns MDS, Hit2 demonstrated stable hydrogen bond interactions with A208 and D270. Among all, Hit3 had the most stable hydrogen bond with all three critical residues; nonetheless, large fluctuations were detected in the case of D270, although the last 100 ns data showed an average threshold distance of 0.35 nm. The detailed H-bond dynamics demonstrated that Hit1 and Hit3 can target all three residues via H-bond interactions; however, there may be a possibility that the H-bond breaks and recovers in the case of T205 and D270.

#### 3.6.5. Principal Component Analysis

**Figure 10:**
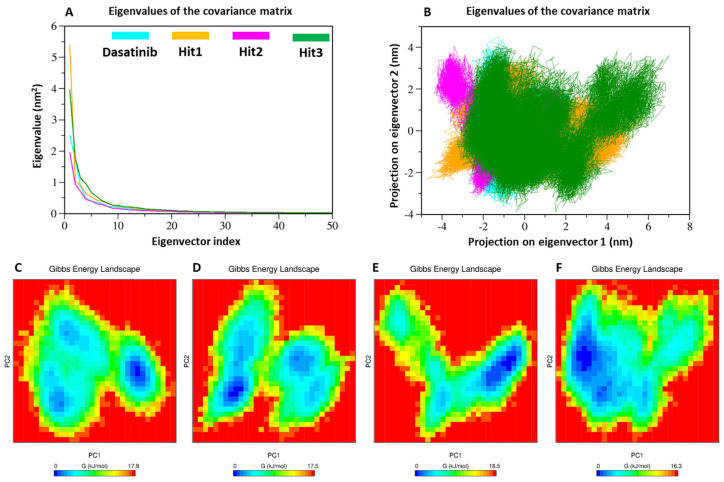
PCA analyses. (**A**) Eigenvector index, (**B**) PC1 and PC2, and (**C**–**F**) free energy landscape of REF, Hit1, Hit2, and Hit3, respectively. The blue spot in the plots indicates the energy minima, whereas the red color indicates a higher energy conformation.

Principal component analysis (PCA) is usually employed to analyze the collective motion of protein–ligand complexes taking backbone or c-alpha atoms [53,54]. The PCA analysis of ACK1-bound ligands reveals that the first few eigenvectors play an important role in the overall motion of all the complexes. The graph plotted in Figure 10A shows the superimposition of the first 50 eigenvectors of the ACK1-Dasatinib, ACK1-Hit1, ACK1-Hit2, and ACK1-Hit3 complexes. The comparative analysis also revealed that the ACK1-Dasatinib complex may display greater conformational variability when compared to hit compounds. It can be noticed from the graph that the first five eigenvectors are responsible for the overall motion of the complexes, accounting for 55.02%, 59.78%, 50.39%, and 57.78% for the ACK1-dasatinib, ACK1-Hit1, ACK1-Hit2, and ACK1-Hit3 complexes, respectively. Interestingly, Hit2 displayed significantly fewer motions when compared to the ACK1-Dasatinib complex; this is then closely followed by ACK1-Hit2 and ACK1-Hit1, indicating their potential as lead candidates. Moreover, we studied the behavior of protein–ligand systems using 2D projection plots of the first two significantly contributing eigenvectors (PC1 and PC2). The superimposition of the plots revealed that all the Hit compounds bound complexes occupying a similar region occupied by the ACK1-Dasatinib complex (Figure 10B). As expected from the eigenvector analysis, Hit2 showed the most stable cluster, followed by Hit3 and Hit1.

#### 3.6.6. Gibbs Free Energy Landscape

Gibbs’s free energy (GFE) landscape was estimated using PC1 and PC2, and the results are plotted in Figure 10C–F. The GFE analysis results revealed that energy values ranged between 0 and 17.9 kJ/mol for REF, 0 and 17.5 kJ/mol for Hit1, 0 and 18.5 kJ/mol for Hit2, and 0 and 16.3 kJ/mol for Hit3. It is clear from the GFE values that Hit1 and Hit3 displayed lower free energy values, and therefore, these complexes may be more thermodynamically stable than Hit2 and REF compounds. The Hit1 and Hit3 GFE plots show a similar pattern with the REF drug, but occupy a more minimum energy state, shown with blue color. In contrast, Hit2 displayed a different pattern of GFE landscape and had a less minimum energy state.

## 4. Discussion

Due to the crucial functions of cellular signaling, kinases have emerged as one of the most intensively studied targets in modern pharmacological research, particularly for cancer [55,56]. ACK1 is a non-receptor tyrosine kinase that is thought to be an oncogene in many tumors and is anticipated to become a therapeutic target [1]. Mahajan et al. reviewed early studies conducted on ACK1 to understand its involvement in cancers such as prostate, breast, pancreatic, ovarian, lung, schwannoma, renal carcinoma, etc. [9]. In the last decades, a number of potential efforts have been conducted to find ACK1 inhibitors. However, none of the inhibitors has reached clinics to date [9,15]. Therefore, there is an urgent need to develop an ACK1-specific inhibitor that can meet clinical requirements. The development of novel drugs against target macromolecules is complex and time consuming. However, this can be sped up using computer-aided drug-designing methods [57,58,59]. Therefore, in the present study, the structure-based pharmacophore modeling approach, one of the most promising in silico techniques for drug design, was selected along with other validation methods, such as MD and MDS (Figure 2). The 3D protein structural databank search reveals that, to date, six ACK1-inhibitors bound X-ray structures were available. Table 1 shows the hydrogen bond interactions of X-ray structure-bound inhibitors with ACK1 active site residues. It can be observed that all the structures display a hydrogen bond with hinge region residue A208. A previous experimental and modeling study revealed that a conventional hydrogen bond with gatekeeper T205 provides better inhibitory efficacy to inhibitors [22]. Only two structures, 3EQR- and 3EQP-bound inhibitors, displayed a hydrogen bonds with these residues (Table 1). The conserved DFG motif residue D270, which plays an important role in the activation of kinase, is observed to interact with 1U4D- and 5ZXB-bound inhibitors [21,45]. The knowledge of the intermolecular interactions of these inhibitors reveals that pharmacophore features of a single structure may not be sufficient to block ACK1 efficiently, and there is a requirement of hybrid PM, which can be built from more than one structure [23,24]. Based on this observation, we selected two structures, 1U4D and 3EQR, which target key residues via hydrogen bond interaction (Table 1). A total of 20 PMs were produced using the *Receptor–Ligand Pharmacophore Generation* protocol of DS (Tables S1 and S2). The generated hypothesis was subsequently merged to obtain a common feature, PM (Figure 3A,B). The pharmacophore features mapping with ACK1 active site residues clearly shows a 1U4D bound ligand map with A208 and D270, whereas 3EQR mapped with A208 and T205. Therefore, in order to obtain desirable HBD feature mapping with T205 and D270, both pharmacophores merged using DS. Finally, a desirable PM was generated (Figure 3C). The three important features, HBA, HBD, and HBD, were mapped with A208, T205, and D270 (Figure 4). We previously developed a ligand-based common feature PM. The model was created using a ligand-based approach, using the five most active inhibitors, including dasatinib, as the training set. PM had five chemical features in total, including two ring aromatics, one hydrophobic, and two hydrogen bond acceptors (HBA) [28]. The two HBA features were able to map A208 and T205. Unfortunately, it was not able to target DFG motif residue D270. In the present investigation, that is why we utilized the knowledge of all the available inhibitor-bound structures and developed a hybrid PM. The current model includes three hydrogen bond acceptor and donor features that might be used to target the three essential residues, T205, A208, and D270. As a result, we believe that by utilizing a hybrid PM, there is a good chance of obtaining hits that target the aforementioned critical residues. The PM was subsequently validated using the GH approach (Table 2) [25]. A drug-like database obtained from four chemical databases utilizing ROF and ADMET descriptor filters was used to screen the PM. Out of the 61,297 compounds, only 866 compounds were mapped to our model. The filtration of a huge number of compounds reveals the significance of PMs in the virtual screening process. We utilized a similar dataset in our earlier pharmacophore-based virtual screening against ACK1, which yielded 3519 compounds [28]. The difference in compound retrieval between the two distinct methods suggests that the structure-based hybrid PM is capable of excluding additional compounds. The obtained compounds were visualized in DS, and the total number was reduced to 351. The final compounds were then subjected to MD using the GOLD program [31]. For comparative analysis, the 1U4D co-crystal ligand, DBQ, and multi-kinase inhibitor drug dasatinib, which have shown nanomolar efficacy against ACK1 in in vitro studies, were docked under similar conditions [16,21]. Our docking results reveal that the docking score of dasatinib was better than the general kinase inhibitor DBQ; therefore, the dasatinib docking score was used as a first filter to select the compounds. The selected dasatinib conformer displayed a Goldscore of 63.78 and a Chemscore of −25.09. The docking complex of dasatinib displayed a hydrogen bond with A208, E206, and S212. Furthermore, hydrophobic interactions with crucial residues T205 and D270 were also observed. Lawrence et al. discovered ACK1 inhibitors utilizing a fragment-based strategy in which they predicted the binding mechanism of the active compound by MD using the GOLD program. The hit compound was shown to form a hydrogen bond with residue A208 in the hinge region. The compound was not observed to form a hydrogen bond with gatekeeper T205 and DFG motif D270, but hydrophobic interactions were observed [50]. Another group discovered selective, orally bioavailable imidazo pyrazine-derived ACK1 inhibitors using virtual screening for the selection of hit compounds. The modeled interactions between the ACK1 and selected hit compounds displayed a hydrogen bond with the gatekeeper T205, and another hit displayed a hydrogen bond with G269 residue close to the DFG motif [47]. Kopecky et al. identified a series of pyrazolo pyrimidines as a novel class of ACK1 inhibitors. Research has also identified the inhibitor-bound structure of PDB id 3EQR and 3EQP. Both structures exhibited a hydrogen bond between the gatekeeper residue T205 and the hinge residue A208. Researchers have demonstrated that the hydrogen bond with the gatekeeper T205 may be responsible for the enhanced anticancer activity of the hit compounds [22]. According to a review of the literature, the hydrogen bond with gatekeeper residue is an essential property observed in several potent kinase inhibitors, including the closely related kinase LCK [60]. According to the aforementioned findings, interaction with key residues, particularly A208, T205, and D270, plays a crucial role in ACK1 protein inhibition. Keeping this in mind, we selected a total of 11 compounds from the MD analysis (Table S3). In structure-based drug design, MDS plays an essential role in the selection of the final drug candidate in computational studies. Usually, simulation runs are performed after docking to check the predicted binding mode stability of the compounds [61,62]. In the present study, the selected compounds were studied under MDS using GROMACS program [35,36]. The simulated compounds were ranked on the basis of BFE, and compounds displaying better binding affinity than dasatinib were selected (Tables S4 and S5) [42]. The stability analysis of the hit compounds was analyzed using commonly used geometrical parameters, such as RMSD and RMSF [32,52]. Figure 7 displays the superimposition of the RMSD and RMSF plots. Our results indicated that hit compounds displayed stable behavior during the 500 ns simulation run, and the average threshold value was <0.3 nm. Moreover, hit compounds displayed strong hydrogen bond-forming potential, suggesting that the inhibitors can interact with proteins with strong affinity (Table S6). The binding affinity was validated using the MM-PBSA method (Table S7) [42]. The average binding free energy from the last 100 ns trajectories revealed that Hit1 displayed significantly better binding affinity with −104.18 kJ/mol than dasatinib −75.42 kJ/mol. The detailed binding mode of the hit compounds DBQ and dasatinib showed that active site key residues were targeted by various types of molecular interactions (Figure 7 and Figure S4). The hinge region residue A208 and DFG motif D270 were targeted via hydrogen bonds by each inhibitor (Figure 7A–C). The desirable hydrogen bond with gatekeeper residue T205 was not observed in the case of Hit2; however, hydrophobic interaction was observed (Figure 7). Moreover, per residue decomposition revealed that residues like R142, K158, G177, T205, N257, and D270 may contribute to polar interactions, whereas L132, V140, A156, M181, I190, M203, L207, L259, and F271 can participate in non-polar interactions (Figure 8). All of these findings are consistent with previously published inhibitor-bound ACK1 crystal structures [21,22,45,46]. The dynamics of the hydrogen bond with critical residues A208, 205, and D270 were also investigated (Figure 9). It was observed that Hit3 can form a stable hydrogen bond with all three residues, which is then followed by Hit1. As expected from the binding mode, Hit2 did not display a hydrogen bond with gatekeeper T205, but the distance analysis revealed that it can form hydrophobic interactions. It is noteworthy to mention that through our modeling work, this is the first time that the ACK1 hit candidate displayed hydrogen bond with three key residues, A208, T205, and D270. We further studied the collective motion of the protein–ligand complexes using PCA analysis [53,54]. The eigenvector index revealed that the first five eigenvectors were responsible for the overall motion of the protein. Covariance analyses revealed that hit compounds occupied less conformational space when compared with dasatinib (Figure 10A,B). The thermodynamic stability of the compounds was also studied using Gibb’s free energy landscapes [35]. The 2D energy plots reveal that Hit3 and Hit1 have minimum free energy values, followed by dasatinib and Hit2 (Figure 10C–F). Finally, we suggest three scaffolds of ACK1 inhibitors as potential platforms for the development of promising anti-cancer inhibitors (Table 5).

## 5. Conclusions

In summary, a structure-based hybrid PM was generated using the known inhibitor-bound crystal structures of ACK1. The PM was observed to be efficient in targeting three key residues of the catalytic domain viz. gatekeeper residue T205, hinge region A208, and DFG motif D270, through desirable hydrogen bond interactions. Four drug-like databases were screened using our PM to obtain potential ACK1 inhibitors. The binding affinity of the compounds was predicted and validated using MD and MDS. The binding free energy analysis was used to rank the simulated complexes, and the results were compared with the multi-kinase inhibitor dasatinib. Binding mode evaluation further reveals that selected ACK1-hit complexes form favorable hydrogen bonds with the gatekeeper residue, hinge region, and DFG motif residues. As a result, we contend that the discovered scaffolds might serve as a new scaffold against ACK1 for anticancer therapies subjected to experimental validation.

## Figures and Tables

**Table 1:** Analysis of the binding pattern of inhibitors with ACK1 based on PDB structures.

PDB ID	Ligands Id	Resolution (Å)	Active Sire Residues							References
			A208	T205	D270	E206	E177	D134	K158	
1U4D	DBQ	2.10	√		√	√		√	√	[21]
3EQR	T74	2.00	√	√						[22]
3EQP	T95	2.00	√	√						
4EWH	T77	2.50	√							[46]
4ID7	1G0	3.00	√			√				[47]
5ZXB	9KO	2.20	√		√		√			[45]

√ denotes hydrogen bond of inhibitor atom with corresponding amino acid.

**Table 2:** The details of pharmacophore validation parameters.

S. No.	Parameters	Calculated Values
1	Total no. of molecules in the database (D)	140
2	Total number of active molecules in the database (A)	20
3	Total number of active molecules in the retrieved hits (Ht)	22
4	Number of retrieved hits by pharmacophore (Ha)	17
5	% Yield of actives [(Ha/Ht) × 100]	77.27%
6	% Ratio of actives [(Ha/A × 100)	85%
7	False-negative [A-Ha]	3
8	False-positive [Ht-Ha]	5
9	Goodness of fit	0.76
10	Enrichment factor (EF)	5.40

**Table 3:** Details of parameters used for the generation of the drug-like database.

Lipinski’s Rule of Five		ADMET Descriptors	
Parameters	Threshold value	Parameters	Threshold value
Number of hydrogen bond donors	≤5	Absorption level	0 (Good)
Number of hydrogen bond acceptors	≤10	Solubility level	3 (Good)
Molecular weight (Da)	≤500	Blood-brain barrier level	3 (Low)
AlogP value	≤5	CYP2D6 prediction	False
		Hepatotoxic prediction	False

**Table 4:** The detailed inter-molecular interactions between Hit compounds and ACK1.

Name	Hydrogen Bond Interactions				Van Der Waals Interactions	π-π/π-Alkyl Interactions
	Amino Acid	Amino Acid Atom	Ligand Atom	Distance (<3.5 Å)		
Hit1	T205	OG1	H28	2.70	D134, L207, S212, N257, G269	L132, V140, A156, K158, M181, I190, M203, L259, F271
	E206	O	H28	1.79		
	A208	HN	O12	2.56		
	R256	O	H35	1.95		
	D270	HN	S13	2.25		
Hit2	E206	O	H32	1.83	G133, G135, K158, T205, L207, G211, N257, G269, F271	L132, V140, A156, M181, I190, L259
	A208	HN	O12	1.74		
	D270	HN	O18	1.93		
Hit3	T205	HG1	O17	2.01	G133, V140, A156, I190, E177, M203, P209, G211, L259, G269, F271	L132, K158, L207, M181
	E206	O	H38	1.73		
	A208	HN	O12	2.30		
	D270	OD2	H42	1.65		
	A208	HN	N8	2.42		
Dasatinib	D270	OD1	H47	2.82	G133, 135, S136, E206, L207, P209, G211, S212	L132, V140, A156, I190, L259
	T205	OG1	H28	2.70		
DBQ	A208	HN	N7	2.48	L132, V140, I190, E206, G211, L207, N257, G269	A156, L259
	D270	HN	N16	2.83		

**Table 5:** The 2D chemical structure of the identified scaffolds and IUPAC names.

Hits	2D Structure	IUPAC Name
Hit1	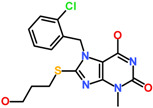	7-[(2-chlorophenyl)methyl]-6-hydroxy-8-(3-hydroxypropylsulfanyl)-3-methyl-purin-2-one
Hit2	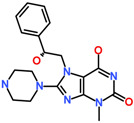	6-hydroxy-7-[(2R)-2-hydroxy-2-phenyl-ethyl]-3-methyl-8-piperazin-1-yl-purin-2-one
Hit3	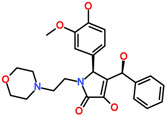	(2S)-4-hydroxy-2-(4-hydroxy-3-methoxy-phenyl)-3-[(S)-hydroxy(phenyl)methyl]-1-(2-morpholinoethyl)-2H-pyrrol-5-one

## Data Availability

Not applicable.

## Supplementary Materials

The following supporting information can be downloaded at: https://www.mdpi.com/article/10.3390/biom13020217/s1, Figure S1: Pharmacophore modeling. (A) Pharmacophore model based on PDB id: 1U4D, (B) and PDB id: 3EQR. The hydrogen bond donor (HBD) and acceptor (HBA) features are shown in pink and green. The cyan and red colors represent hydrophobic (HYP) and pos ionizable (PI) features, respectively. Figure S2: The 3D crystal structure of ACK1 PDB id: 1U4D is used for molecular docking. (A) The docking site was defined near the bound ligand. (B) The overlay of the selected compounds after molecular docking analysis in the side active site of ACK1. Figure S3: RMSD analysis of the selected Hit candidates, co-crystalized ligand DBQ, and dasatinib after 500 ns MDS study. Figure S4: The 3D and 2D binding modes of DBQ and dasatinib (shown with cyan stick) inside the active site of ACK1. The upper panel of image A-B) displaying the binding mode of DBQ and the lower panels C-D) displaying Dasatinib. The hydrogen bonds are shown with dark green dashed lines. The van der Waals and pi-alkyl interactions were shown with light green, purple, and pink colored spheres. Table S1: Pharmacophore models generated from PDB: 1U4D. Table S2: Pharmacophore models generated from PDB: 3EQR. Table S3: List of potential compounds obtained after molecular docking. A total of 11 compounds showed a better Goldscore and Chemscore than the reference inhibitor. Table S4: Details of binding free energy and H-bond interactions of simulated compounds after 50 ns MD simulations. Table S5: Details of the molecular dynamics simulation system prepared for a 500 ns run. Table S6: Details of molecular docking and molecular dynamics simulation analysis after 500 ns of selected Hit compounds. Table S7: Binding free energy components of Hit compounds and dasatinib calculated from the MM-PBSA method.
