# Supplementary material for: Identification of Activated Cdc42-Associated Kinase Inhibitors as Potential Anticancer Agents Using Pharmacoinformatic Approaches
Source: Biomolecules. 2023 Jan 22;13(2):217. doi: 10.3390/biom13020217 (PMC9953130; doi:10.3390/biom13020217)
Supplement: Supplementary file 1 [file biomolecules-13-00217-s001.zip › biomolecules-2135829-supplementary.pdf]

biomolecules

MDPI

Article (Supplementary File)

Identification of Activated Cdc42-Associated Kinase Inhibitors as Potential Anticancer Agents Using Pharmacoinformatic Approaches

Vikas Kumar, Raj Kumar, Shraddha Parate, Danishuddin, Gihwan Lee, Moonhyuk Kwon, Seong-Hee Jeong, Hyun-Su Ro, Keun Woo Lee and Seon-Won Kim

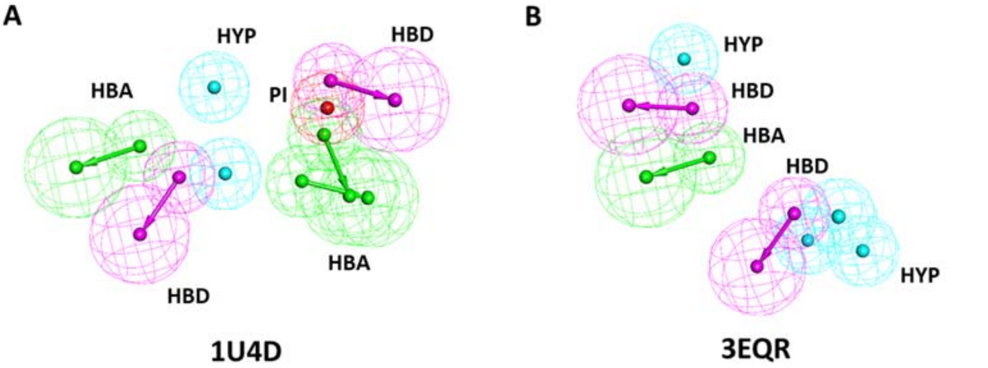

Figure S1. Pharmacophore modeling. A) Pharmacophore model based on PDB id: 1U4D, B) and PDB id: 3EQR. The hydrogen bond donor (HBD) and acceptor (HBA) features were shown in pink and green colors. The cyan and red color resenting hydrophobic (HYP) and pos ionizable (PI) feature respectively.

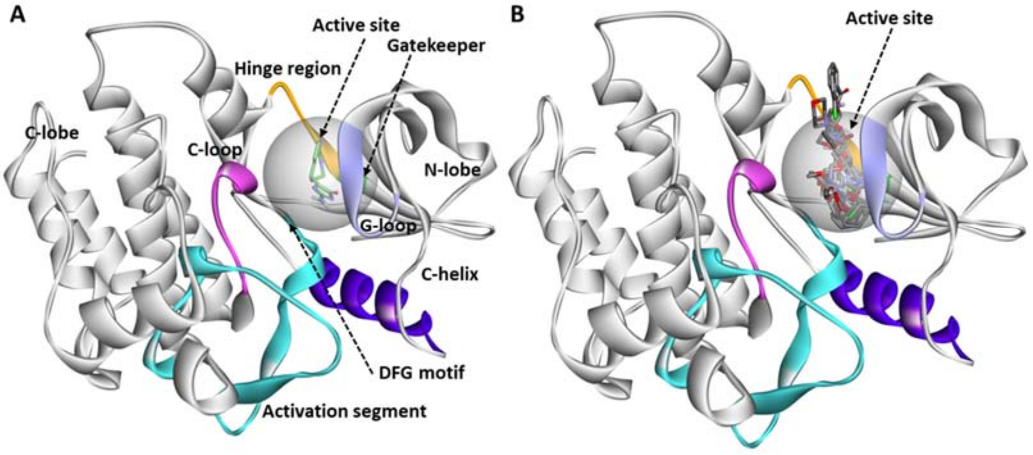

Figure S2. The 3D X-ray structure of ACK1 PDB id: 1U4D is used for molecular docking. **A)** The docking site was defined near the bound ligand. B) The overlay of the selected compounds after molecular docking analysis in the side active site of ACK1.

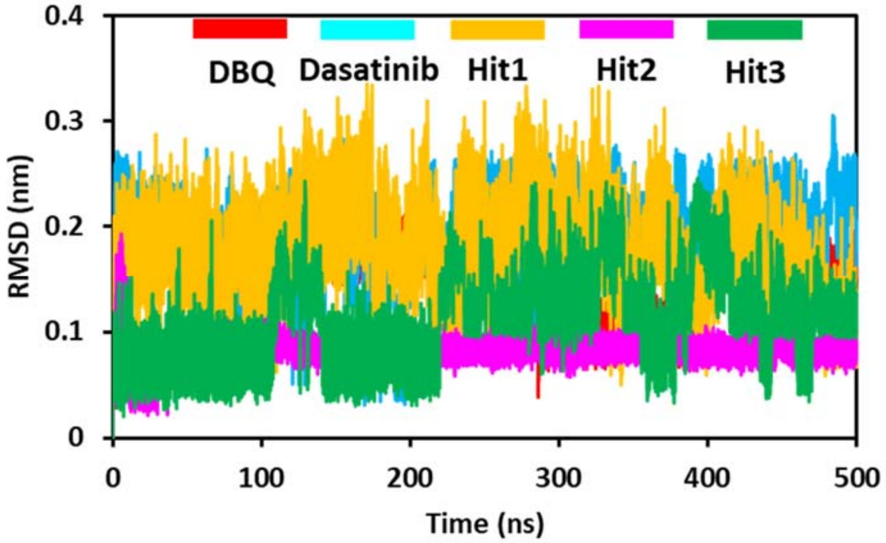

Figure S3. RMSD analysis of the selected Hit candidates, co-crystalized ligand DBQ, and dasatinib after 500 ns MDS study.

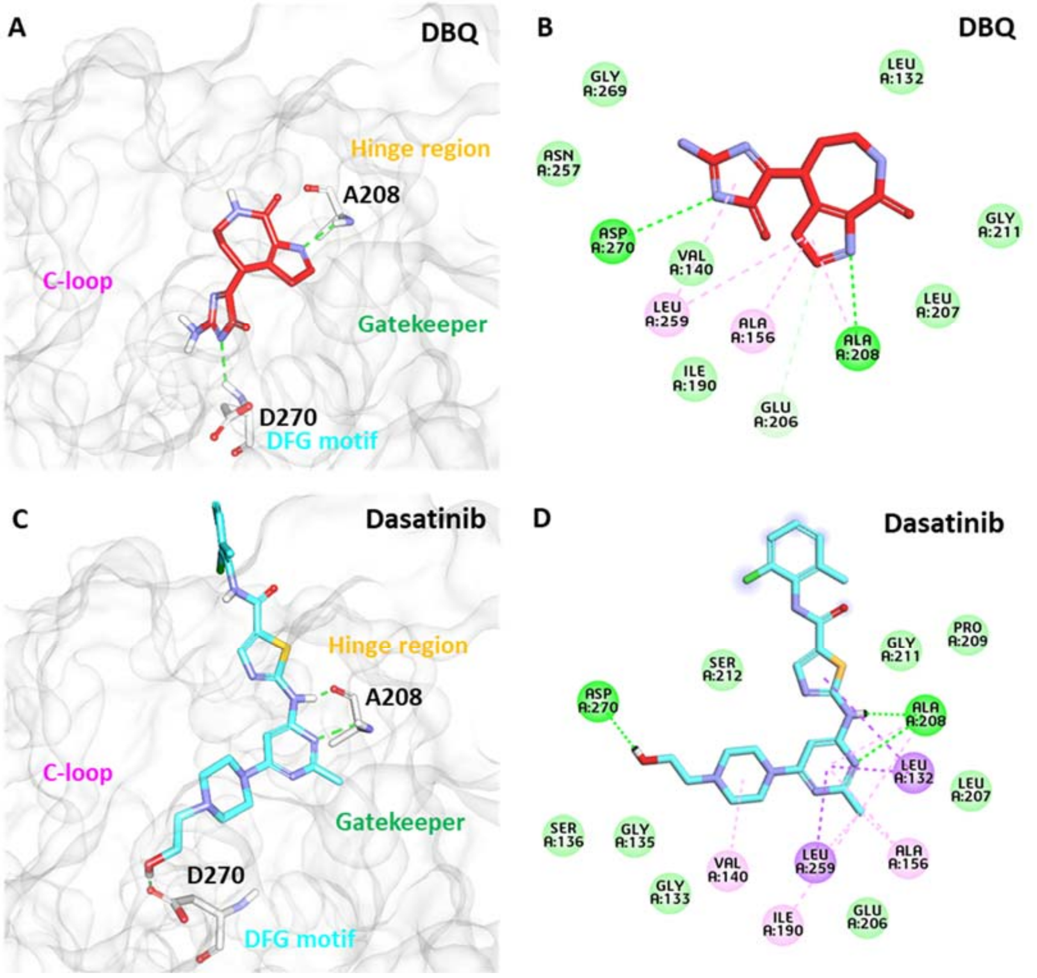

Figure S4.The 3D and 2D binding modes of DBQ and dasatinib (shown with cyan stick) inside the active site of ACK1. The upper panel of image A-B) displaying the binding mode of DBQ and the lower panels C-D) displaying Dasatinib. The hydrogen bonds were shown with dark green dashed lines. The van der Waals and pi-alkyl interactions were shown with light green, purple, and pink color spheres.

Table S1. Pharmacophore models generated from PDB: 1U4D.

**Pharmacophore models**			
Pharmacophore	Number of Features	Feature Set	Selectivity Score
Pharmacophore 01	6	AADHHP	11.498
Pharmacophore 02	6	AADDHH	11.018
Pharmacophore 03	6	AAADDH	11.018
Pharmacophore 04	6	AADDHH	11.018
Pharmacophore 05	6	AADDHH	11.018
Pharmacophore 06	6	AAADDH	11.018
Pharmacophore 07	6	AAADHH	10.104
Pharmacophore 08	6	AAADHH	10.104
Pharmacophore 09	5	ADHHP	9.9830
Pharmacophore 10	5	AADHP	9.9830

Table S2. Pharmacophore models generated from PDB: 3EQR.

**Pharmacophore Summary**			
Pharmacophore	Number of Features	Feature Set	Selectivity Score
Pharmacophore 01	6	ADDHHH	11.841
Pharmacophore 02	6	DDHHHH	11.841
Pharmacophore 03	6	ADDHHH	11.841
Pharmacophore 04	6	ADDHHH	11.841
Pharmacophore 05	6	ADDHHH	11.841
Pharmacophore 06	6	ADHHHH	10.927
Pharmacophore 07	6	ADHHHH	10.927
Pharmacophore 08	5	ADDHH	10.326
Pharmacophore 09	5	DDHHH	10.326
Pharmacophore 10	5	DDHHH	10.326

Table S3. List of potential compounds obtained after molecular docking. A total of 11 compounds show better Goldscore and Chemscore than the reference inhibitor.

**Name**	**Goldscore**	**Chemscore**	**2D**
Compound 1	69.90	-27.37	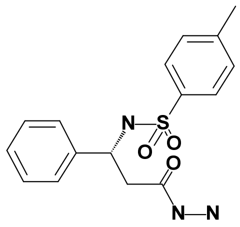
Compound 2	68.37	-27.58	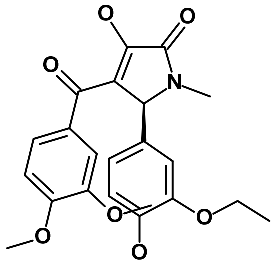

Compound 3	66.69	-27.00	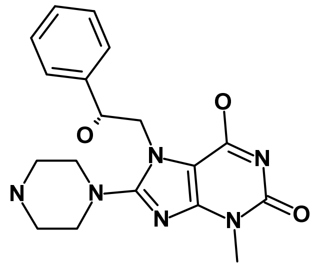
Compound 4	66.02	-28.01	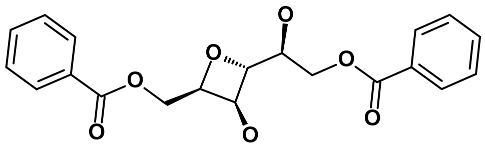
Compound 5	64.70	-25.48	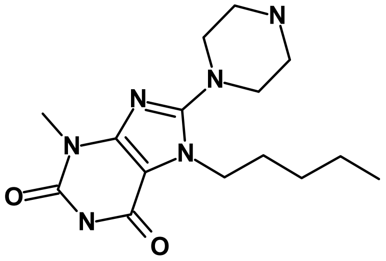
Compound 6	64.59	-31.05	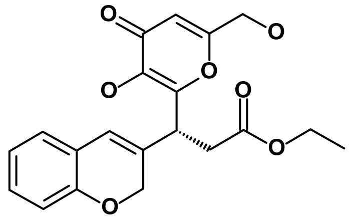
Compound 7	64.24	-27.45	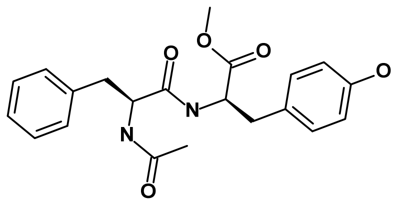
Compound 8	63.96	-28.48	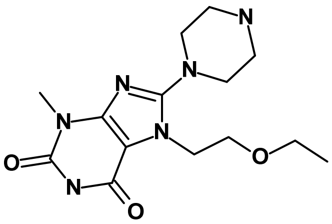
Compound 9	63.91	-26.63	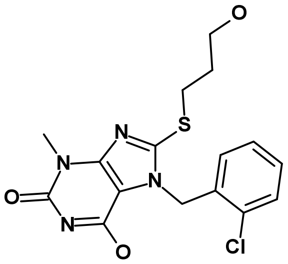

Compound 10	63.83	-28.25	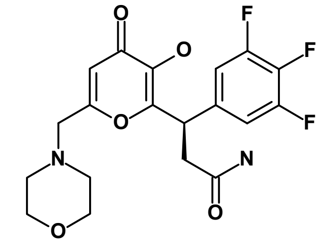
Compound11	63.80	-31.14	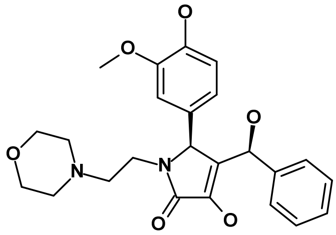
Dasatinib	63.78	-25.09	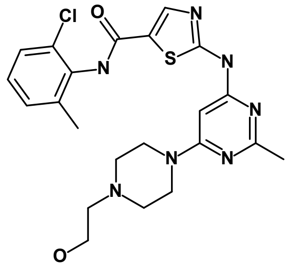
DBQ	45.48	-22.33	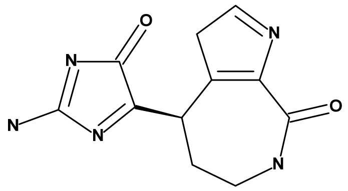

Table S4. The details of binding free energy and H-bond interactions of simulated compounds after 50 ns MD simulations.

Name	Binding free energy	H-bond interactions
Compound 1	-28.41	E206
Compound 2	-53.40	P209, S212
Compound 3	-86.81	D134, E206, A208, G269
Compound 4	-76.98	A208
Compound 5	-75.87	D134, T205, E206, A208
Compound 6	-62.36	A208
Compound 7	-12.11	K158, A208, D270
Compound 8	-64.99	D134, T205, E206, A208
Compound 9	-93.35	T205, E206, A208, N257
Compound 10	-63.64	E206, A208, D270
Compound11	-76.12	T205, E206, A208, D270

Table S5. The details of the molecular dynamics simulation system prepared for 500 ns run.

System	No. of TIP3P water molecules	No. of Na+ ions
ACK1 + Hit 1	12324	2
ACK1 + Hit 2	12325	2
ACK1+ Hit 3	12327	2
ACK1 + Dasatinib	12332	2
ACK1-DBQ	12325	2
1U4D_Apo	12325	2

Table S6. The details of molecular docking and molecular dynamics simulation analysis after 500 ns of selected Hit compounds.

Name	Goldscore	Chemscore	Binding free energy	MDS analysis			
				RMSD (protein)	RMSD (ligand)	RMSF (protein)	H-bond
Hit 1	63.91	-26.63	-104.18	0.19	0.17	0.08	2.75
Hit 2	66.69	-27.00	-86.31	0.17	0.08	0.09	3.23
Hit 3	63.80	-31.14	-80.23	0.20	0.11	0.11	2.62
Dasatinib	63.78	-25.09	-75.42	0.18	0.19	0.10	1.84
DBQ	45.48	-22.33	-43.62	0.18	0.15	0.10	1.83
IU4D-Apo	-	-	-	0.24	-	0.11	-

Table S7. Binding free energy components of Hit compounds and dasatinib calculated from MM-PBSA method.

Inhibitors	van der Waals (kJ/mol)	Electrostatic (kJ/mol)	Polar solvation (kJ/mol)	SASA energy (kJ/mol)	Binding energy (kJ/mol)
Hit1	-195.08+/-12.71	-50.67+/-12.64	161.19+/-16.62	-19.61+/-0.92	-104.18+/-15.99
Hit2	-197.56+/-11.23	-59.89+/-6.73	191.65+/-15.38	-20.51+/-0.69	-86.31+/-13.60
Hit3	-169.48+/-14.17	-75.40+/-15.79	184.78+/-25.04	-20.12+/-1.28	-80.23+/-14.71
Dasatinib	-162.93+/-11.45	-36.68+/-12.13	141.93+/-26.71	-17.74+/-1.25	-75.42+/-17.31
DBQ	-119.37+/-13.31	-51.26+/-21.70	141.04+/-39.11	-13.96+/-1.04	-43.62+/-18.49
